# Naturally Occurring Yeasts Associated with *Thaumatotibia leucotreta* Can Enhance the Efficacy of the Cryptophlebia Leucotreta Granulovirus

**DOI:** 10.3390/pathogens12101237

**Published:** 2023-10-13

**Authors:** Marcel van der Merwe, Michael D. Jukes, Caroline Knox, Sean D. Moore, Martin P. Hill

**Affiliations:** 1 Department of Biochemistry and Microbiology, Rhodes University, P.O. Box 94, Makhanda 6140, South Africa; m.jukes@ru.ac.za (M.D.J.); caroline.knox@ru.ac.za (C.K.); 2 Centre for Biological Control, Department of Zoology and Entomology, Rhodes University, P.O. Box 94, Makhanda 6140, South Africa; seanmoore@cri.co.za (S.D.M.); m.hill@ru.ac.za (M.P.H.); 3 Citrus Research International, P.O. Box 5095, Walmer, Gqeberha 6065, South Africa

**Keywords:** synergism, baculoviruses, biological activity, biological control

## Abstract

Yeasts associated with lepidopteran pests have been shown to play a role in their survival, development, and oviposition preference. It has been demonstrated that combining these yeasts with existing biological control agents can enhance their efficacy. The tortricid *Thaumatotibia leucotreta* is a phytosanitary pest in the South African citrus industry, with the baculovirus Cryptophlebia leucotreta granulovirus (CrleGV) being one of the components that can control this pest. Several yeast species were shown to be associated with *T. leucotreta* larvae, which affected their behaviour and development. A series of detached fruit bioassays were performed to determine whether the combination of yeast with CrleGV enhances its efficacy. These assays included determining the optimal yeast/virus ratio, testing all isolated yeast species in combination with CrleGV, and further improving yeast/virus formulation by adding an adjuvant. The optimal yeast concentration to use alongside CrleGV was determined to be 10^6^ cells·mL^−1^. *Pichia kluyveri*, *P. kudriavzevii*, *Kluyveromyces marxianus*, and *Saccharomyces cerevisiae* in combination with CrleGV reduced larval survival compared to CrleGV alone. The addition of molasses and BREAK-THRU^®^ S 240 to *P*. *kudriavzevii* and *S*. *cerevisiae* in combination with CrleGV did not notably improve their effectiveness; however, there was an observed decrease in larval survival. In future studies, field trials will be conducted with combinations of CrleGV and *P. kudriavzevii* or *S*. *cerevisiae* to investigate whether these laboratory findings can be replicated in orchard conditions.

## 1. Introduction

*Thaumatotibia leucotreta* (Meyrick) (Lepidoptera: Tortricidae) (false codling moth) is a significant pest of the South African citrus industry and other citrus-producing regions throughout sub-Saharan Africa [1]. The feeding of larvae on fruit causes damage, potentially reducing yields and resulting in financial losses [2]. Improved management practices in recent years have effectively suppressed *T. leucotreta* populations in South African orchards [3]. However, *T. leucotreta* is now primarily a phytosanitary risk rather than a destructive crop pest, with zero tolerance in the European Union and other export markets [4].

Several control options are available to manage *T. leucotreta*, including chemical, biological, cultural, and pheromone-based methods [3,4]. Treatments are strategically combined into an integrated pest management (IPM) programme to suppress *T. leucotreta*, beginning early in the season [5,6]. IPM programmes are considered the most effective approach to managing this pest and rely on the intelligent selection and implementation of pest monitoring and control options. Biological control agents are fast becoming an integral part of IPM programmes due to the stringent regulations around chemical insecticides, which limit their use and availability [7]. A number of biological agents are available for use against *T. leucotreta* in South Africa, most notably baculoviruses [3,8].

The baculovirus Cryptophlebia leucotreta granulovirus (CrleGV) has been extensively used for more than 15 years to control *T. leucotreta* throughout South Africa [3,9]. Despite the significant benefits of baculoviruses, they have a few drawbacks, including their sensitivity to ultraviolet (UV) degradation, slower speed of kill, short field persistence and narrow host range [8,9,10,11].

While CrleGV is an effective biological control agent against *T. leucotreta* [9], continued research and innovation are necessary to enhance its efficacy. Improving the effectiveness of baculoviruses is crucial for their long-term use as biopesticides. The principal biological limitation affecting the efficacy of baculoviruses is the possibility for them to be ingested by their intended host before penetrating the fruit [12]. Efforts to improve the performance of baculoviruses have mainly focused on increasing virus exposure time to larvae before they penetrate the fruit through the use of attractants and feeding stimulants [9,13]. Adding adjuvants such as molasses to virus formulations has been shown to significantly improve virus efficacy compared to applying the virus alone [9,14]. Recently, the incorporation of mutualistic microorganisms associated with the target pest into formulations has been proposed as a new larval attractant [15].

Previous attempts to enhance Cydia pomonella granulovirus (CpGV) performance through the combination of feeding stimulants and larval attractants have not yielded definitive improvements [13,16,17]. These efforts have predominantly centred around host plant volatiles that attract *Cydia pomonella* (Linnaeus) (Lepidoptera: Tortricidae) neonates, but they have primarily appeared to facilitate host location rather than stimulate feeding behaviour [18,19]. Microbes consumed and harboured within the gut of insects have the potential to profoundly influence both the survival and behaviour of their host [20]. The significance of microbial communities in insect–plant interactions is indispensable [21,22]. *Cydia pomonella* larvae have a close affiliation with yeasts from the genus *Metschnikowia,* which were isolated from their gut and feeding galleries. Larval feeding assays demonstrated that *M. andauensis* positively impacted *C*. *pomonella* larvae, accelerating their development and lowering mortality rates. Volatiles produced by *M*. *andauensis* also elicited upwind flight in adult *C*. *pomonella* females, which resulted in an increased number of eggs being oviposited on apples that had been inoculated with yeast [15]. The combination of yeasts, isolated from *C*. *pomonella* larvae, with CpGV occlusion bodies (OBs) noticeably improved the virus’s efficiency, both in controlled laboratory experiments and in practical field applications [23]. Yeasts from the genus *Metschnikowia* actively promoted larval feeding and facilitated the ingestion of CpGV [15,23]. Furthermore, considering the widespread availability of *Saccharomyces cerevisiae* in commercial use, its combination with CpGV was also evaluated. Larval mortality recorded in CpGV assays using *S*. *cerevisiae* closely resembled that of yeasts isolated from *C*. *pomonella* larvae [14].

A recent study aimed to identify yeast species present within the digestive tract of *T. leucotreta* larvae collected from citrus orchards across South Africa. This led to the identification of six yeast species: *Meyerozyma guilliermondii*, *Pichia kluyveri*, *Pichia kudriavzevii*, *Hanseniaspora uvarum*, *Clavispora lusitaniae*, and *Kluyveromyces marxianus* [24]. Larval development assays demonstrated that *M*. *guilliermondii*, *H*. *uvarum*, and *P*. *kluyveri* accelerated larval development and reduced mortality, while *P*. *kluyveri*, *H*. *uvarum*, *P. kudriavzevii*, and *K*. *marxianus* were shown to influence the feeding preference of neonate *T. leucotreta* in larval feeding assays. Additionally, the oviposition preference of adult *T. leucotreta* females was affected by *M*. *guilliermondii*, *P. kudriavzevii*, and *H*. *uvarum*, with an increased number of eggs being oviposited on Navel oranges inoculated with these yeasts.

Yeast strains hold promising potential as abundant sources of novel biological agents that can be harnessed for the augmentation and improvement of established control agents. In this study, we aimed to determine whether previously isolated yeast species associated with *T. leucotreta* larvae increase the efficacy of CrleGV when combined. Furthermore, the addition of an adjuvant (molasses) to improve the yeast/virus mixture efficacy was analysed.

## 2. Materials and Methods

### 2.1. Thaumatotibia leucotreta Culture

*Thaumatotibia leucotreta* eggs were obtained from the heterogeneous culture, known as “Mixed Colony”, held at Rhodes University’s Department of Zoology and Entomology, South Africa. Eggs were stored in Petri dishes sealed with parafilm in a 25 °C controlled environment (CE) room with a relative humidity of 30–60%. Once the eggs had turned dark brown, a piece of cotton wool moistened with double distilled water (ddH_2_O) was placed in the Petri dish to ensure that emerging *T. leucotreta* neonates did not dehydrate before being used.

### 2.2. Detached Fruit Bioassays

Three sets of detached fruit bioassays were conducted to (i) determine the optimal yeast concentration to use in combination with CrleGV, (ii) assess the effectiveness of combining each of the isolated yeasts with CrleGV, and (iii) evaluate the efficacy of the yeast/virus mixture through the addition of an adjuvant and surfactant. CrleGV was applied at an LC_50_ concentration of 9.31 × 10^7^ OBs·mL^−1^ for all treatments [25]. The LC_50_ concentration was selected as this would result in 50% mortality of the *T. leucotreta* population.

Batches of Navel oranges were collected from orchards in the Sunday’s River Valley in the Eastern Cape Province of South Africa. No postharvest treatments had been applied to the oranges. The Navel oranges were stored in a 4 °C cold room to preserve the fruit until use. The oranges were checked weekly, with fruit showing any sign of disease or mould being discarded. Oranges were stored for a maximum period of 6–8 weeks. Navel oranges were removed from 4 °C cold storage one day prior to being used in a detached fruit bioassay. They were inspected for any sign of disease or mould before being thoroughly washed in a 0.5% bleach solution (*v*/*v*), rinsed twice in ddH_2_O, and allowed to air dry in a 25 °C CE room overnight.

Yeast cultures were grown in a Yeast extract Peptone Dextrose (YPD) medium containing 40 units·mL^−1^ of penicillin (Pen) and 40 µg·mL^−1^ of streptomycin (Strep) (Thermo Fisher Scientific, Waltham, MA, USA) for 20 h at 27 °C while shaking. Cell counts were adjusted appropriately with ddH_2_O.

A modified version of the detached fruit bioassay described by Moore et al. [25] was used to determine the effectiveness of combining CrleGV with yeast against *T. leucotreta*. The first set of bioassay treatments included a ddH_2_O control, CrleGV alone, *P. kudriavzevii* at 2 × 10^8^ cells·mL^−1^ plus CrleGV, *P. kudriavzevii* at 2 × 10^6^ cells·mL^−1^ plus CrleGV, and *P. kudriavzevii* at 2 × 10^4^ cells·mL^−1^ plus CrleGV. A yeast concentration of 10^8^ cells·mL^−1^ was selected as a starting point based on prior research on yeast/virus synergism in *C*. *pomonella* [23]. *Pichia kudriavzevii* was selected as the yeast isolate to use during these bioassays due to its attractiveness to *T. leucotreta* neonates and adult females [24]. The second set of detached fruit bioassays evaluated the efficacy of combining *M*. *guilliermondii*, *P*. *kluyveri*, *H*. *uvarum*, and *K*. *marxianus* with CrleGV. *Saccharomyces cerevisiae* was also included as a treatment, as it forms part of the artificial diet on which *T. leucotreta* larvae are reared [26]. Yeasts were applied at a concentration of 2 × 10^6^ cells·mL^−1^ with CrleGV at 9.31 × 10^7^ OBs·mL^−1^, as it was previously shown to be the optimal yeast/virus ratio. Finally, detached fruit bioassays were conducted to enhance the efficacy of the yeast/virus mixture by adding molasses and BREAK-THRU^®^ S 240, as these have been shown to enhance the efficacy of the virus in field trials [9]. *Pichia kudriavzevii* was selected because it has been shown to influence *T. leucotreta* neonates and adult female behaviour [24]. *Saccharomyces cerevisiae* was included, as it is a commercially available yeast strain and has previously been shown to enhance the efficacy of CpGV [14]. The third set of bioassay treatments included a ddH_2_O control, CrleGV, *P. kudriavzevii* (at 2 × 10^6^ cells·mL^−1^) plus CrleGV, and *S*. *cerevisiae* (at 2 × 10^6^ cells·mL^−1^) plus CrleGV, each with an adjuvant (molasses) and surfactant (BREAK-THRU^®^ S 240) (Evonik Industries AG, Essen, Germany) at 0.25% and 0.005%, respectively.

Navel oranges were placed onto a sterile metal rack and sprayed with a specific treatment until runoff using a handheld sprayer. The treated oranges were subsequently positioned on a platform with none of the fruit coming into contact with each other, transported to a 25 °C CE room and allowed to dry for 30–45 min. Five *T. leucotreta* neonates were placed on each fruit. Thirty Navel oranges were used per treatment, with each treatment replicated three times. Detached fruit bioassays were run for 14 days, after which the Navel oranges were dissected and inspected for the presence or absence of live *T. leucotreta* larvae.

### 2.3. Statistical Analysis

A generalized linear mixed model (GLMM) was utilised to determine whether the inclusion of yeast to CrleGV impacted *T. leucotreta* larval survival. The data from the three experiments were individually analysed, as each experiment was conducted sequentially. The GLMM was specified using a binomial error distribution and a logit link function. Significant differences in larval survival between treatments were assessed using likelihood ratio tests. Where significant differences were found, pairwise comparisons were performed using the “emmeans” R package and were adjusted for multiple comparisons using Tukey adjustment [27]. All statistical analyses were performed using R version 4.4.2 (R Core Team 2022) and all models were fitted using the “glmmTMB” R package [28]. Graphs were produced using GraphPad Prism version 10.0.3.

## 3. Results

### 3.1. Optimising the Yeast/Virus Ratio

**Figure 1:**
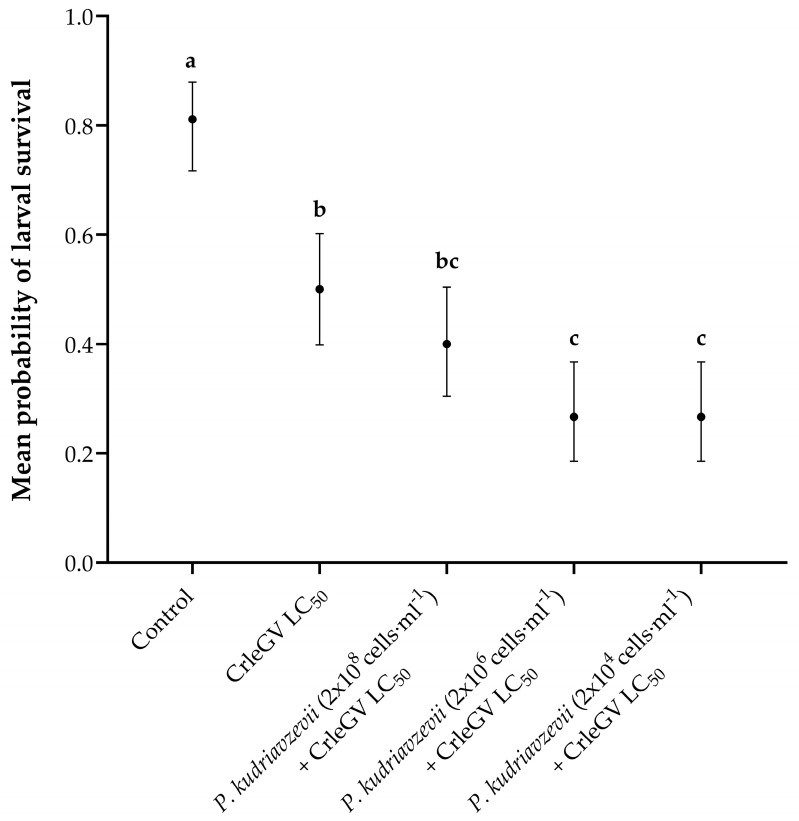
*Thaumatotibia leucotreta* larval survival in 14-day detached fruit bioassays (*n* = 3). Navel oranges were treated with ddH_2_O, CrleGV at 9.31 × 10^7^ OBs·ml^−1^, and *P*. *kudriavzevii* at varying concentrations ranging from 2 × 10^8^ to 2 × 10^4^ cells·mL^−1^ plus CrleGV. Different letters indicate statistically significant differences (*p* ≤ 0.05).

Detached fruit bioassays were conducted to determine the optimal yeast concentration to apply alongside CrleGV. There was evidence for a statistically significant difference in larval survival between treatments (X^2^ = 62.525, df = 4, *p* < 0.001). The application of *P*. *kudriavzevii* at a concentration of 2 × 10^8^ cells·mL^−1^ (beta = 0.405, z-value = 1.346, *p* = 0.6623) in combination with CrleGV did not result in a significant reduction in larval survival when compared to the use of CrleGV alone. However, when *P*. *kudriavzevii* was applied at concentrations of 2 × 10^6^ cells·mL^−1^ (beta = 1.012, z-value = 3.179, *p* = 0.0129) and 2 × 10^4^ cells·mL^−1^ (beta = 1.012, z-value = 3.179, *p* = 0.0129), it led to a notable decrease in larval survival by 23.33% (Figure 1). All treatments were significantly different from the ddH_2_O control treatment.

### 3.2. Combining CrleGV with Yeast

**Figure 2:**
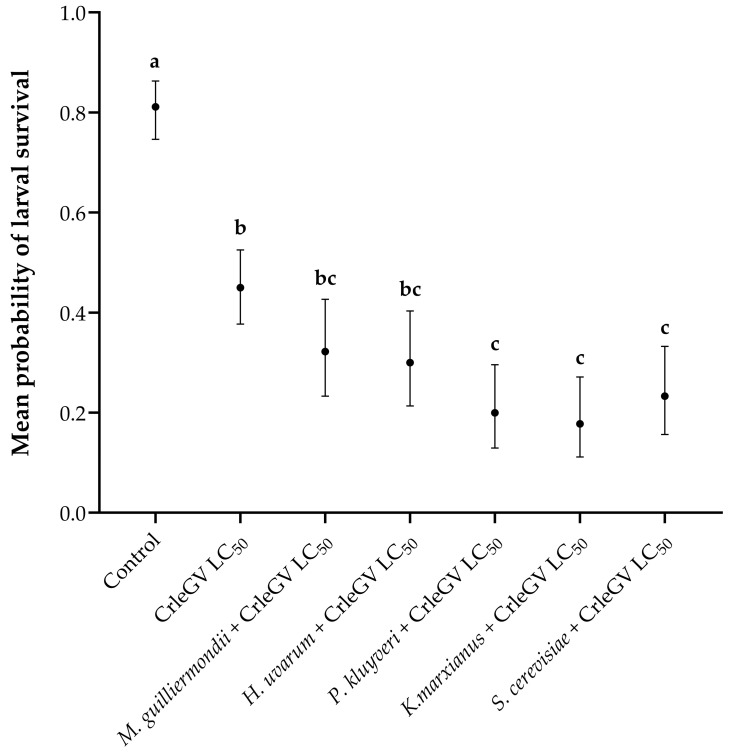
Larval survival of *T. leucotreta* in 14-day detached fruit bioassays (*n* = 3). Navel oranges were treated with *H*. *uvarum*, *K*. *marxianus*, *M. guilliermondii*, *P*. *kluyveri*, and *S*. *cerevisiae* combined with CrleGV. Different letters indicate statistically significant differences (*p* ≤ 0.05).

There was evidence for a statistically significant difference in larval survival between treatments (X^2^ = 143.82, df = 6, *p* < 0.001). In comparison to the use of CrleGV alone, the inclusion of *P*. *kluyveri*, *K*. *marxianus*, and *S*. *cerevisiae* alongside the virus resulted in reductions in larval survival by 25.01% (with beta = 1.187, z-value = 3.913, *p* = 0.0018), 27.24% (with beta = 1.332, z-value = 4.243, *p* = 0.0004), and 21.68% (with beta = 0.990, z-value = 3.402, *p* = 0.0119), respectively (Figure 2). *Meyerozyma guilliermondii* (beta = 0.543, z-value = 2.006, *p* = 0.4108) and *H*. *uvarum* (beta = 0.647, z-value = 2.357, *p* = 0.2174) did not significantly reduce larval survival when applied in combination with CrleGV compared to the virus alone. All treatments were significantly different from the ddH_2_O control treatment.

### 3.3. Enhancing the Efficacy of Yeast/Virus Formulation

**Figure 3:**
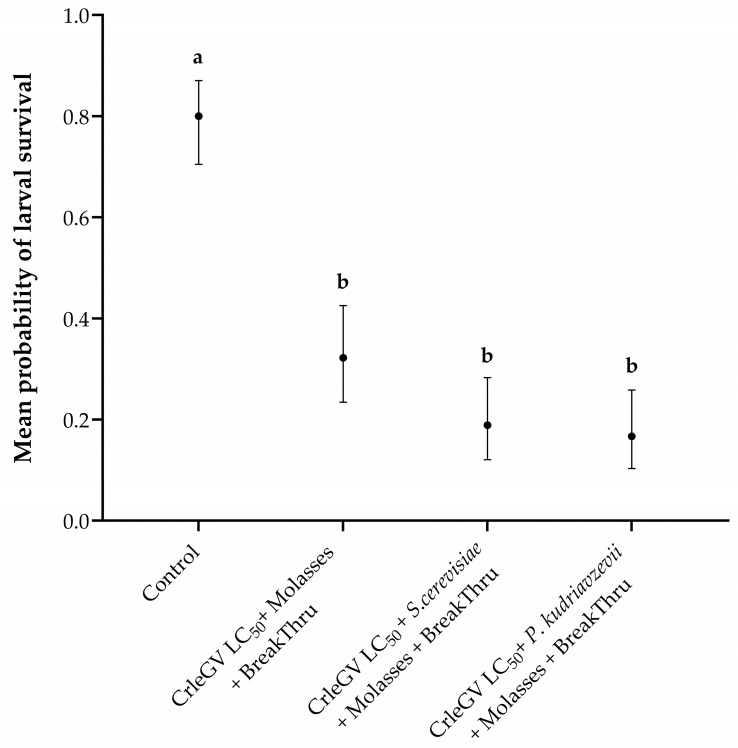
*Thaumatotibia leucotreta* larval survival in 14-day detached fruit bioassays including molasses and BREAK-THRU^®^ S 240 (*n* = 3). Navel oranges were treated with ddH_2_O, CrleGV alone, *P*. *kudriavzevii* plus CrleGV, and *S*. *cerevisiae* plus CrleGV, with yeasts applied at 2 × 10^6^ cells·mL^−1^ and CrleGV at 9.31 × 10^7^ OBs·mL^−1^. Except for the control, all treatments had the addition of molasses and BREAK-THRU^®^ S 240 at 0.25% and 0.005%, respectively. Different letters indicate statistically significant differences (*p* ≤ 0.05).

There was evidence for a statistically significant difference in larval survival between treatments (X^2^ = 80.059, df = 3, *p* < 0.001). The addition of molasses and BREAK-THRU^®^ S 240 did not significantly enhance the efficacy of *P*. *kudriavzevii* (beta = 0.866, *z*–value = 2.393, *p* = 0.0783) or *S*. *cerevisiae* (beta = 0.714, z-value = 2.032, *p* = 0.1763) formulations compared to CrleGV alone (Figure 3). The inclusion of molasses and BREAK-THRU^®^ S 240 resulted in a 15.56% and 13.34% decrease in larval survival, respectively, when *P*. *kudriavzevii* and *S*. *cerevisiae* were present, compared to treatments without these additives. Furthermore, no significant differences were recorded between the two yeast isolates (beta = −0.152, z-value = −0.390, *p* = 0.9799). All treatments were significantly different from the ddH_2_O control treatment.

## 4. Discussion

The influence of mutualistic yeast on insect behaviour and development has been studied recently [15,20,24,29,30]. Yeasts have demonstrated their vital role as a nutritional foundation for the growth of insect larvae and in their capacity to impact the feeding patterns and behaviour of newly hatched larvae [30,31,32,33]. Additionally, volatile compounds produced by yeasts evoke significant behavioural responses in insects [31,34,35]. *Cydia pomonella* larvae have a strong symbiotic relationship with yeast belonging to the genus *Metschnikowia* [15]. These yeasts have been demonstrated to play a critical role in promoting the growth of *C*. *pomonella* larvae by providing vital nutrients and safeguarding them against fungal infections, thereby reducing mortality [15]. The interactions between insect pests and their mutualistic microbes present an ideal target for manipulation and use in biological control. However, incorporating beneficial microbes to enhance larval feeding with existing biological control agents has been limited [14,23].

Previous research showed improved effectiveness of baculovirus treatments utilising mutualistic yeast at 10^8^ cells·mL^−1^ [14,23]. However, no significant differences were recorded in this study when *P*. *kudriavzevii* was applied in mixtures with CrleGV at a similar concentration [36]. The concentration at which *P*. *kudriavzevii* was applied may have resulted in an unfavourable yeast/virus ratio and resulted in yeast cells not being thoroughly covered with CrleGV. Detached fruit bioassays using a 100-fold serial dilution of *P*. *kudriavzevii*, ranging from 2 × 10^8^ to 2 × 10^4^ cells·mL^−1^, were set up to determine the optimal yeast/virus ratio. Significant differences were recorded when using the lower yeast concentrations of 10^6^ cells·mL^−1^ and 10^4^ cells·mL^−1^ alongside CrleGV. The effectiveness of a biological control agent utilising microorganisms relies on how much of the agent used reaches the target pest [37]. Hence, the use of lower yeast/virus ratios may result in greater uptake of CrleGV OBs, assuming a consistent intake of contaminated yeast cells during larval feeding. An overabundance of yeast can also lead to high alcohol levels, which could negatively affect insect physiology and behaviour [38].

Previous studies have shown that neonate *T. leucotreta* exhibited altered behaviour when exposed to *H*. *uvarum*, *P*. *kluyveri*, *P*. *kudriavzevii*, and *K*. *marxianus* [24]. Specifically, they displayed an attraction to yeast broth that had been inoculated with these yeast species for feeding. It was also demonstrated that the mortality rate of *T. leucotreta* larvae significantly decreased when Navel oranges were treated with *M*. *guilliermondii*, *P*. *kluyveri*, *H*. *uvarum*, and *S*. *cerevisiae* [24]. These characteristics render these yeasts remarkably well-suited as potential feeding stimulants for *T. leucotreta*. The association of *S*. *cerevisiae* with several key agricultural pests has been extensively documented [39,40,41]. The addition of *S*. *cerevisiae* to CpGV resulted in a level of larval mortality comparable to that of the wild-type isolate (*Metschnikowia pulcherrima*) associated with *C*. *pomonella* [14]. *Saccharomyces cerevisiae* was included here, due to its commercial availability and inclusion in the artificial diet used to rear *T. leucotreta* [26]. A significant decrease in larval survival was recorded with most gut-associated yeasts and *S*. *cerevisiae*. However, *M*. *guilliermondii* and *H*. *uvarum* did not enhance the efficacy of CrleGV. The positive effects of their ingestion on the development and survival of *T. leucotreta* larvae may have reduced the effectiveness of CrleGV at the LC_50_ dose [24].

Once *T. leucotreta* larvae penetrate the fruit’s rind, they are unlikely to ingest any additional OBs [25]. Molasses has been used as a larval-feeding stimulant with some success in improving baculovirus efficacy [9,13]. Furthermore, owing to the adhesive properties of molasses, it may unintentionally lead to an increase in the number of OBs adhering to the fruit’s surface. BREAK-THRU^®^ S240 was included as it decreases the surface tension of spray droplets, leading to enhanced retention and deposition of spray treatments [42]. Additionally, it exhibits a “super-spreading” effect that significantly enhances the coverage of surfaces by spray treatments, resulting in improved dispersion of spray residues [42]. Adding molasses and BREAK-THRU^®^ S240 to *P*. *kudriavzevii* and *S*. *cerevisiae* plus CrleGV treatments did decrease larval survival but not significantly, compared to that of the virus alone. The efficacy of CrleGV could be further enhanced by adding molasses to yeast/virus formulations, as it has previously been demonstrated to be an effective adjuvant [9,13,14]. It might be necessary to fine-tune the ratio of molasses and BREAK-THRU^®^ S240 utilized alongside *P*. *kudriavzevii* and *S*. *cerevisiae* to achieve the ideal working conditions. An LC_50_ concentration of CrleGV was selected, as this would result in 50% mortality of the *T. leucotreta* population. Thus, an increase or decrease in mortality could be observed when combining CrleGV with a specific treatment. The mortality rates of *T. leucotreta* observed in detached fruit bioassays using the LC_50_ concentration of CrleGV were comparable to previously reported rates [25]. As previously found with *C*. *pomonella* [14,23], the addition of mutualistic yeast to a virus treatment proved effective in increasing larval mortality.

The inclusion of yeast isolated from *T. leucotreta* to CrleGV formulations proved effective in increasing virus efficacy. Additionally, the inclusion of molasses and BREAK-THRU^®^ S240 further increased the formulation’s effectiveness, compared to CrleGV being applied alone. Taken together, the results of this study indicate that *P. kudriavzevii* and *S*. *cerevisiae* hold potential for use in biocontrol, especially when combined with other well-established control techniques for use against *T. leucotreta*. These yeasts could potentially serve as a supplement to enhance larval feeding and thus virus ingestion, and as a distinctive approach for pest monitoring and attraction. Future work will entail conducting field trials with *P*. *kudriavzevii* and *S*. *cerevisiae* to determine whether their ability to increase the effectiveness of CrleGV in laboratory assays can be replicated in citrus orchards.

## Data Availability

The data presented in this study are available on request from the corresponding author.
